# Health Impact Assessment of Sulfolane on Embryonic Development of Zebrafish (*Danio rerio*)

**DOI:** 10.3390/toxics7030042

**Published:** 2019-08-23

**Authors:** Soham M. Shah, Michael Wahba, Linlong Yu, Gopal Achari, Hamid R. Habibi

**Affiliations:** 1 Department of Biological Science, University of Calgary, Calgary, AB T2N 1N4, Canada; 2 Department of Civil Engineering, University of Calgary, Calgary, AB T2N 1N4, Canada

**Keywords:** sulfolane, toxicity, morphometrics, behaviour, gene expression, zebrafish

## Abstract

Sulfolane is a widely used polar, aprotic solvent that has been detected by chemical analysis in groundwater and creeks around the world including Alberta, Canada (800 µg/mL), Louisiana, USA (2900 µg/mL) and Brisbane, Australia (4344 µg/mL). Previous research provided information on adverse effects of sulfolane on mammals, but relatively little information is available on aquatic organisms. This study tested the effects of sulfolane (0–5000 µg/mL) on early development of zebrafish larvae, using various morphometric (survival, hatching, yolk sac and pericardial oedema, haemorrhaging, spinal malformations, swim bladder inflation), growth (larval length, eye volume, yolk sac utilisation), behavioural (touch response, locomotor activity and transcript abundance parameters (*ahr1a*, *cyp1a*, *thraa*, *dio1*, *dio2*, *dio3*, *11βhsd2*, *gr*, *aqp3a*, *cyp19a1b*, *ddc*, *gria2b* and *hsp70*) for 120 h. Embryos were chronically exposed to sulfolane throughout the experimental period. For locomotor activity, however, we also investigated acute response to 2-h sulfolane treatment. Sulfolane sensitivity causing significant impairment in the observed parameters were different depending on parameters measured, including survival (concentrations greater than 800 µg/mL), morphometric and growth (800–1000 µg/mL), behaviour (500–800 µg/mL) and transcript abundance (10 µg/mL). The overall results provide novel information on the adverse health impacts of sulfolane on an aquatic vertebrate species, and an insight into developmental impairments following exposure to environmental levels of sulfolane in fish embryos.

## 1. Introduction

Sulfolane has a number of synonyms: tetramethylene sulfone, bondelane A and 2,3,4,5-tetrahydrothiophene-1,1-dioxide to name a few. It is a stable, clear, colourless liquid organosulphur with a high polarity, making it highly soluble in water (1,266,000 mg/L at 20 °C [1]. It was first developed by the Shell Oil Company in the 1940s to aid in the separation of aromatics from hydrocarbons in the gasoline boiling range and H_2_S, CO_2_, COS and CS_2_ extraction in natural gas sweetening [2,3,4]. Today, however, sulfolane use has spread to numerous multinational oil and gas corporations as well as to multiple different applications and industries that include fractionation of wood tars, production of paint, synthetic resins, food products, plastics, soaps, pharmaceuticals, insecticides, electronics and polymers [5,6,7]. Though sulfolane utilisation remains primarily in the oil and gas industry, increased global production, transportation and use of sulfolane by multiple industries has resulted in increased problems with storage, leaks, spills and leaching from disposal sites; all of which have resulted in environmental sulfolane contamination of water and soil [8]. 

In Alberta, Canada, concentrations of sulfolane have been recorded as high as 800 µg/mL in groundwater [9] and 270 µg/mL in certain creeks [10]. In Louisiana, USA, concentrations have been documented as high as 2900 µg/mL in groundwater and 126 µg/mL in surface water [11]. In Brisbane, Australia, concentrations ranging from 3153 µg/mL to 4344 µg/mL of sulfolane have been reported in groundwater [12]. Soil sulfolane contamination has also been recorded as high as 3400 mg/kg [13]. All of these reports clearly show the widespread unintended presence of sulfolane in the environment, despite efforts by corporations in certain areas, to minimise sulfolane release into the environment. Of approximately 150 sulfolane production and utilising sites worldwide [14,15], a number of locations operate under no environmental regulations. Given the extensive use of sulfolane, unintended release into the environment is inevitable. Unfortunately, sulfolane degradation is slow at low temperatures and only occurs under ideal conditions, which when combined with a high solubility in water, makes sulfolane prone to long-distance transport and a high likelihood for the spread of contamination to large areas and numerous living organisms [8,16]. 

Current environmental guidelines for sulfolane in Canada are based on studies using terrestrial model organisms, including rats, mice, guinea pigs and rabbits, as well as some aquatic organisms. In rats and mice, sulfolane exposure was associated with increased respiratory rate, responsiveness to auditory stimuli, rigid tails and rear leg shaking [6,17,18]. Exposure to sulfolane reduced serum alkaline phosphatase, isocitrate dehydrogenase, glutamic-oxaloacetic transaminase and glutamic-pyruvic transaminase [6,18]. In aquatic organisms, however, sulfolane toxicity information is mainly limited to LC_50_ concentrations for fathead minnows (*Pimephales promelas*, >1000 µg/mL), rainbow trout (*Oncorhynchus mykiss*, 1264 µg/mL), goldfish (*Carassius auratus*, 4800 µg/mL), mosquito fish (*Gambusia sp.*, 1930 µg/mL) and stickleback (*Gasterosteus sp.*, 1760 µg/mL) following 24 h to 7 days exposure [19,20,21,22,23]. Apart from the adverse impact of sulfolane exposure on survival, no other adverse impacts of sulfolane have been reported in aquatic vertebrates. For aquatic invertebrates, sideswimmers (*Hyalella azteca*) have a sulfolane LC_50_ concentration of 1516 µg/mL following 96 h exposure [20]. In *Daphnia manga*, LC_50_ concentrations range from 40 µg/mL to 3274 µg/mL following 48 h sulfolane exposure [19,22,23]. Further studies by the Canadian Council of Ministers of the Environment (CCME) reported the LC_50_ concentration for *Daphnia manga* as 1245 µg/mL [24]. A study on *Ceriodaphnia dubia* also demonstrated decreases in reproductive success following sulfolane exposure of 375 µg/mL for 7 days [20]. These results as well as more recent findings by Agency for Toxic Substances and Disease Registry (ATSDR) [25] have contributed in part to development of environmental guidelines, although there is uncertainty regarding guidelines for aquatic environments as indicated in the documents published by Health Canada and CCME [26,27].

Therefore, further studies would be necessary to provide more information on toxicity of sulfolane in aquatic organisms. The present study investigated the adverse health impact of sulfolane using zebrafish (*Danio rerio*) as a suitable aquatic vertebrate model organism that meets the OECD guidelines for the testing of environmental contaminants [28]. Zebrafish embryos provide additional benefits such as ease of rearing, rapid development, transparency and endogenous feeding (lecithotrophic) until 120 h post fertilization (hpf) [29,30,31]. We used morphometric, behavioural and transcript abundance to study dose-related effects of sulfolane. More specifically, we measured survival, hatching, morphometric characteristics (yolk sac and pericardial oedemas, haemorrhaging, spine malformations and swim bladder inflation) and growth (larval length, eye volume, yolk sac utilisation) as suitable parameters based on previous studies [32,33,34,35]. The behavioural assessment was based on response to touch stimuli and locomotor activity following two different sulfolane exposure durations on zebrafish larvae to provide an insight into potential developmental neurotoxicity of sulfolane as suggested previously [36,37,38,39]. The transcript abundance assessment provides an understanding of the sublethal impacts of sulfolane exposure and an insight into potential mechanisms of action for sulfolane. Overall, the present study provides support for the hypothesis that sulfone at concentrations found in the environment exerts adverse health impacts on developing zebrafish embryos, and an insight into morphological, behavioural and molecular changes resulting from exposure to this contaminant. 

## 2. Materials and Methods 

### 2.1. Zebrafish Husbandry and Embryo Collection

Zebrafish were raised in an Aquatic Habitat System for zebrafish (ZF0601; Aquatic Habitats, Apopka, FL, USA) equipped with an automated water quality monitor and control system which maintained water at 28 °C, 7.5 pH and 800 µS conductivity under a 14:10 hour light–dark cycle. Other parameters measured and maintained were chlorine, total gas pressure, O_2_, CO_2_, nitrogenous compounds (ammonia, nitrite and nitrate) and hardness. Fish were fed adult zebrafish complete diet (Zeigler Bros., Inc., Gardners, PA, USA) twice a day. For breeding, one-year old zebrafish in approximately 1:1 male to female ratio were kept overnight in breeding chambers on the same zebrafish system. The following morning, zebrafish embryos were collected, plated (10 embryos to a well in a 12-well plate), and treated within 2 h with various treatments in 3 mL autoclaved fish system water (28 °C; pH: 7.5; conductivity: 800 µS). Autoclaved fish system water (AW) was used as a control in this study for all assessments. All protocols and procedures were approved by the University of Calgary Animal Care Committee (protocol #AC15-0183) on 1 March 2016 and followed the guidelines set forth by the Canadian Council of Animal Care.

### 2.2. Sulfolane Treatment

Sulfolane (99%; Sigma-Aldrich Canada Co., Oakville, ON, Canada) was dissolved in AW to create a stock sulfolane solution (SS) of 5000 µg/mL. The SS was stored in a cool (4 °C), dark, dry place for a maximum of 2 weeks. Appropriate amounts of SS and AW were added to the embryos within 2hpf in each well of a 12-well plate to achieve the desired exposure concentrations in a total volume of 3 mL. Exposures were classified into 2 types: chronic and acute sulfolane exposures. Chronic effect was investigated by exposing zebrafish embryos to continuous treatment with different concentrations of sulfolane (0, 1, 5, 10, 25, 50, 75, 100, 125, 150, 200, 250, 400, 500, 600, 800, 1000, 2000, 3000, 4000 and 5000 µg/mL), followed by assessing morphometric, behavioural and transcript abundance at 72 or 120 hpf. Acute effect was investigated by rearing zebrafish embryos in AW only (no sulfolane exposure: 0 µg/mL) until 72 or 120 hpf, followed by treatment with sulfolane (0, 100, 200, 400, 800 and 1600 µg/mL) for 2 h. The 12-well plates were incubated at 28 °C. Partial water changes (66%) were carried out every day to ensure minimal concentration of waste products (urea and CO_2_) up to assessment time. Morphometric assessment was carried out by transferring the embryos into AW following anaesthesia as described below immediately after treatments.

A concentration check of sulfolane was performed following all assessments (Table 1) using a liquid–liquid extraction and gas chromatography (GC) protocol established by Khan et al [40]. Sulfolane measurement was carried out by extracting 1 mL samples collected from the wells, using 1.5 mL dichloromethane (DCM, ≥99.9%, Sigma-Aldrich Canada Co., Oakville, ON, Canada). Vials containing the 1 mL of sample and 1.5 mL of DCM were placed in a shaker (200 rpm) for 30 min before transferring the aqueous layer into a GC vial. GC was performed using Agilent 6890 Gas Chromatography machine (Agilent Technologies, Santa Clara, CA, USA) equipped with an auto-sampler and Flame Ionisation Detector (FID; Table 1). Pure helium (99.999%, Praxair Canada Inc., Calgary, AB, Canada) at a pressure of 250 kPa was used as a carrier gas and the injection volume and temperature of injection port were set to 1.00 µL and 165 °C, respectively. The system was operated on split-less mode. The initial temperature of the oven was set to 90 °C and was ramped up to 175 °C at a rate of 10 °C/min where it was held constant for 3 min. The FID detector temperature was set to 250 °C. The detection limit for GC-FID was 1 ppm or 1 µg/mL. 

### 2.3. Morphometric Assessment

The morphometric assessment was completed using a modified protocol established by Fraysse et al [32]. Chronically exposed zebrafish larvae to various concentration of sulfolane (0, 50, 100, 150, 200, 400, 500, 600, 800, 1000, 2000, 3000, 4000 and 5000 µg/mL) were removed from each well at 72 and 120 hpf and transferred into MS222 (Tricaine mesylate from Sigma-Aldrich Canada Co., Oakville, ON, Canada) anesthetising solution before imaging. In the present study, we used a low concentration of MS222 (2.64 µg/mL) for sedation to avoid stress and other extraneous effects based on previous studies [41,42,43]. A higher concentration of MS222 (264 µg/mL) was used for euthanasia. Three larvae were individually positioned dorsoventrally in a 3.2 mm cavity slide with 2.5% methylcellulose (Sigma-Aldrich Canada Co., Oakville, ON, Canada) for imaging, using a dissecting microscope (M165FC, Leica Microsystems Inc., Concord, ON, Canada). The larvae were transferred using a large eye dropper to avoid any physical damage. Following imaging, zebrafish larvae were placed in AW for 10 min to remove the effects of anaesthetic and placed back into the respective wells of the 12-well plate. Thus, the same larvae were used to assess growth characteristics at 120 hpf. A water change (~100%) was carried out following the morphometric assessment. 

Larvae were assessed for survival (*n* = 10–15 wells, each containing 10 embryos) and morphometric parameters (yolk sac oedema, pericardial oedema, haemorrhaging, spinal malformations and swim bladder malformations; *n* = 8–15 wells, each containing 10 embryos) at 72 and 120 hpf. Hatching of the zebrafish embryos to become larvae was also documented at 48, 72 and 120 hpf (*n* = 10–15 wells, each containing 10 embryos). All these parameters were measured with a binary choice (presence or absence) and were selected based on previous studies reflecting the effect of a wide range of mechanisms upon chemical exposure [32,33,34]. Survival was assessed by the presence of a heartbeat and was normalised to 100% for control (no sulfolane exposure: 0 µg/mL) zebrafish larvae. Successful hatching was considered when the chorion was completely detached from the embryo. Yolk sac oedema was characterised by the presence of fluid above or below the yolk sac, resulting in the distention of the yolk sac cavity. Pericardial oedema was characterised by the inflation of the cavity to twice its size due to the presence of fluid. Haemorrhaging was characterised by the pooling of blood in a tissue. Frequency of spinal malformation was also determined for all treatments. Swim bladder malformation was only measured at 120 hpf and identified when larvae lacked an inflated swim bladder.

Growth parameters, including larval length, horizontal and vertical eye diameter, and yolk sac width were quantified at 72 and 120 hpf (*n* = 36–40 individual larvae) using the Leica Application Suite 4.5 (Leica Microsystems Inc., Concord, ON, Canada) to obtain measurements. All measurements were taken in millimetres. Larval length was measured following straightening of the larvae as best as possible in methylcellulose. The horizontal and vertical eye diameters were averaged and converted to eye volume based on the assumption that the eye is a sphere [35] and the yolk sac width was converted to percent utilisation by establishing the width at 120 hpf for control (no sulfolane exposure—0 µg/mL) zebrafish larvae as 100% yolk sac utilisation. Deceased embryos and larvae were not imaged.

### 2.4. Behavioural Assessment

Response to touch stimuli was assessed in zebrafish larvae at 120 hpf (*n* = 8–15 wells, each containing 10 embryos) following chronic exposure to sulfolane. The assay was based on response to touch stimuli by a blunt needle, and the data was recorded as a binary choice (presence or absence). No movement following stimuli was recorded as an absence of touch response. The result is expressed as frequency of surviving larvae that responded to touch stimuli following exposure to varying concentrations (0–5000 µg/mL) of sulfolane. 

Locomotor response was determined in zebrafish larvae following acute and chronic exposure to sulfolane (*n* = 30–40 individual larvae). Locomotor activity was induced by exposing larvae to light and dark using a ZebraBox (ViewPoint Behaviour Technology, Montréal, QC, Canada) as described previously [39]. Acute sulfolane treatment consisted of exposure of randomly chosen 120 hpf larvae to varying concentrations of sulfolane (0, 100, 200, 400, 800 and 1600 µg/mL) for a period of 2 h. This was done by placing one 120 hpf zebrafish larva in each well of the 48-well plate and adding appropriate amounts of SS (total volume 1 mL). Chronic treatment was characterised by continuous exposure of zebrafish larvae to sulfolane (0, 100, 200, 400, 800 and 1600 µg/mL) up to 120 hpf. Locomotor activity tracking of zebrafish larvae in response to light cycling (10 min light/10 min dark) following one-hour dark incubation was achieved by contrast imaging using ZebraLab (ViewPoint Behaviour Technology, Montréal, QC, Canada) software. A ratio of the total distance travelled while in the dark over the total distance travelled while in the light was determined in each case. The ratio was then normalised to 100% for control zebrafish larvae (0 µg/mL) and statistics were run. The results are plotted as percentage of locomotor activity relative to control.

### 2.5. Transcript Abundance

For every dose of sulfolane tested, 4 wells containing 10 embryos each (total of 40 embryos) were plated following embryo collection. At 120 hpf, all surviving larvae exposed to the same concentration of sulfolane were pooled together in a petri dish. Ten zebrafish larvae were then randomly selected, and flash frozen in liquid nitrogen in 2 mL Eppendorf tubes for RNA extraction. These steps were repeated 4 times using different breedings (*n* = 4). RNA extraction was completed using the protocol established by Peterson et al. [44] using TRIzol^®^ reagent (Fisher Scientific Co., Ottawa, ON, Canada). However, to maximise RNA extraction yield, an overnight RNA precipitation was completed using RNA grade glycogen (Fisher Scientific Co., Ottawa, ON, Canada). RNA was then reconstituted in dindiethylpyrocarbonate (DEPC, Fisher Scientific Co., Ottawa, ON, Canada) water and RNA levels quantity and quality were quantified by measuring the absorbance at 260 nm and 230 nm using a NanoDrop 2000 (Fisher Scientific Co., Ottawa, ON, Canada). A DNAse I treatment was carried out post quantification to remove remnant DNA. cDNA synthesis was then completed following manufacturer’s protocol using a high-capacity cDNA reverse transcription kit (Fisher Scientific Co., Ottawa, ON, Canada).

Following cDNA synthesis, a (q)RT-PCR was completed using SsoFast EvaGreen Supermix (Bio-Rad Laboratories (Canada) Ltd., Mississauga, ON, Canada) and primers from the University of Calgary DNA Synthesis Lab (Calgary, AB, Canada) in a CFX96 Touch™ Real-Time PCR Detection System (Bio-Rad Laboratories (Canada) Ltd., Mississauga, ON, Canada). The genes were selected based on results generated from the morphometric and behavioural assessment studies, and include aryl hydrocarbon receptor (*ahr1a*; NM_131028); cytochrome p450 (*cyp1a*; NM_131879); thyroid receptor (*thraa*; NM_131396); deiodinases 1, 2, and 3 (*dio1*; NM_001007283, *dio2*; NM_212789, *dio3*; NM_001177935); 11 beta–dehydrogenase 2 (*11βhsd2*; NM_212720); glucocorticoid receptor (*gr*; EF567112); aquaporin (*aqp3a*; NM_213468); brain aromatase (*cyp19a1b*; NM_131642); dopa decarboxylase (*ddc*; NM_213342); AMPA-type glutamate receptor (*gria2b*; NM_131895) and heat shock protein (*hsp70*; AF210640). The transcript abundance was measured at 120 hpf. The sequences of the primer sets used, their annealing temperatures and their respective efficiencies for the PCR are shown in Supplementary Data Table S1. The efficiency of the primer sets was between 93.6% and 100.5% and was determined by completing serial dilutions of the cDNA. Specificity of the primer sets was confirmed by the presence of a single peak in a melting curve analysis. An initial dose range of 125 to 4000 µg/mL sulfolane exposure was completed; however, if the lowest dose 125 µg/mL was significantly different from control (0 µg/mL), an expanded dose range was completed from 1 to 4000 µg/mL. The PCR thermocycling parameters were as follows; initial activation at 95 °C for 2 min, followed by 40 cycles of 10 seconds at 95 °C, 30 seconds of the annealing temperature (51–66.7 °C) and 10 seconds at 95 °C. PCR products underwent a final elongation at 72 °C for 10 min. Expression of the genes of interest was normalised to the abundance of the housekeeping gene, *ß-actin* (NM_181601) using the formula 2^−ΔΔCt^.

### 2.6. Statistics

All statistics were completed using R (Version 3.4.3) with an alpha value of 0.05 and were graphed using Prism 7 software (GraphPad Software Inc., San Diego, CA, USA). Normal distribution and homogeneity of the data were tested, using Shapiro–Wilk W test and Levene’s tests respectively. A nonparametric independent samples test, Kruskal−Wallis and a post hoc Conover–Iman all pairs comparison test for multiple comparisons (PMCMRplus package [45]) were used since the observations did not meet requirements of normality and homoscedasticity required for a parametric test. Additionally, for morphometric results showing greater than 50% response at the highest concentration tested (5000 µg/mL), the effective dose giving 50% maximal response (EC_50_) was estimated using a nonlinear regression (least squares fit). All results are presented as means ± standard error of mean (S.E.M) except for the nonlinear regression which is expressed as mean and confidence interval (CI: lower limit − upper limit). Dissimilar letters on data points indicate significant differences among treatments (*p* < 0.05).

## 3. Results 

### 3.1. Morphometric Assessment

#### 3.1.1. Survival and Hatching

**Figure 1:**
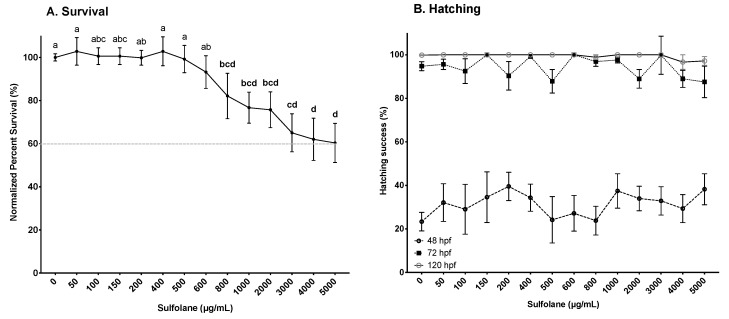
Dose-related effect of sulfolane exposure in µg/mL on survival at 120 hpf (**A**) and percent hatching at 48, 72 and 120 hpf (**B**) of zebrafish (*Danio rerio*) larvae. Survival was based on the presence of heartbeat while successful hatching was considered when the chorion was completely detached from the embryo. The points represent mean and SEM. The dashed line indicates percent survival at the maximum sulfolane exposure concentration. Unmatched letters represent statistical significance (*p* < 0.05) based on tests with Conover–Iman all pairs comparison test. *n* = 10–15 wells, each containing 10 embryos.

Survival was determined by the presence of a heartbeat. Exposure to sulfolane for 120 hpf, resulted in a gradual decrease in survival (*p* = 0.00023; Kruskal−Wallis test). However, significant differences from control were observed in the groups exposed to sulfolane concentrations higher than 800 µg/mL (*p* < 0.033; Conover–Iman all pairs comparison test; Figure 1A). The lowest survival (60.4 ± 9.1%) was observed at the maximum tested sulfolane concentration of 5000 µg/mL. Thus, no estimation of the EC_50_ was carried out as the mortality was less than 50%. In this study, we also tested the effects of sulfolane on hatching. Exposure to sulfolane was without effect on hatching (48 hpf: *p* = 0.63, 72 hpf: *p* = 0.28, 120 hpf: *p* = 0.32; Kruskal−Wallis test). All surviving embryos were hatched between 48 and 72 hpf (Figure 1B). Results shown represent hatching in the surviving embryos only.

#### 3.1.2. Yolk Sac Utilisation

**Figure 2:**
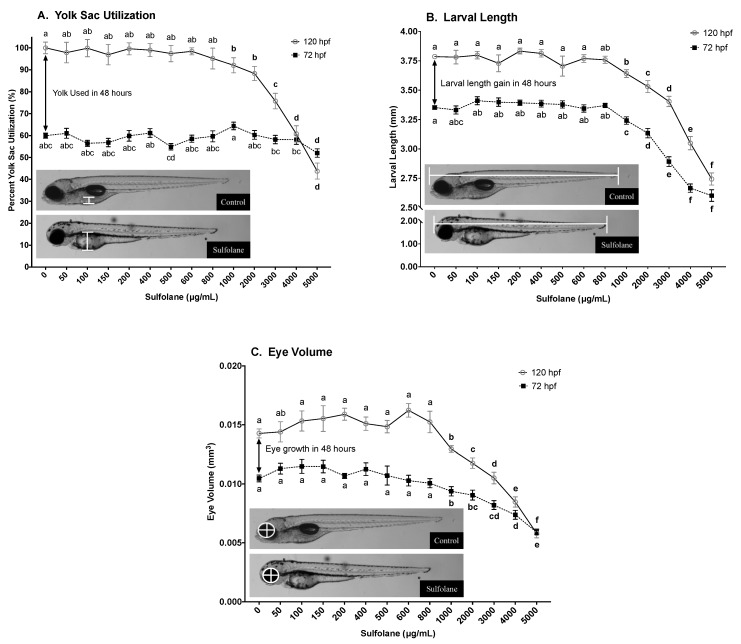
Dose-related effect of sulfolane exposure in µg/mL on the yolk sac utilisation (**A**), larval length (**B**) and eye volume (**C**) quantified in zebrafish (*Danio rerio*) larvae at 72 and 120 hpf. Yolk sac width was converted to percent utilisation by establishing the width at 120 hpf for control (0 µg/mL) zebrafish larvae as 100% yolk sac utilisation. Larval length was measured from the tip of the head to the tip of the tail. The horizontal and vertical eye diameters were averaged and converted to eye volume based on the assumption that the eye is a sphere [35]. The points represent mean and SEM. Unmatched letters represent statistical significance (*p* < 0.05) based on tests with Conover–Iman all pairs comparison test. *n* = 36–40 individual larvae.

Yolk sac width was quantified following continuous sulfolane exposure of 72 and 120 hpf. Relative yolk sac utilisation of sulfolane exposed and control zebrafish larvae was then calculated by equating yolk sac width of control zebrafish larvae at 120 hpf as 100% yolk sac utilisation. As a result, yolk sac utilisation of control zebrafish larvae at 72 hpf was found to be 60.0 ± 1.2% with respect to yolk sac utilisation of the control fish at 120 hpf (Figure 2A). Exposure to sulfolane for 120 hpf resulted in dose-related reduction in yolk sac utilisation (72 hpf: *p* < 0.0001; 120 hpf: *p* < 0.0001; Kruskal−Wallis test) and significant differences from control were observed at concentrations greater than 1000 µg/mL (*p* < 0.025; Figure 2A) with a Conover–Iman all pairs comparison test. At 72 hpf, a small but significant reduction was observed only at 5000 µg/mL (*p* < 0.0001; Conover–Iman all pairs comparison test; Figure 2A). The yolk sac utilisation within the 48 h between 72 and 120 hpf can be calculated by the difference between 72 and 120 hpf plots on the graph; i.e., 40% of the yolk is used for control zebrafish larvae within those 48 h. However, upon sulfolane exposure, there was a decrease in the amount of yolk used (*p* < 0.0001; Kruskal−Wallis test) between 72 and 120 hpf for doses greater than 3000 µg/mL (*p* < 0.026; Conover–Iman all pairs comparison test; Figure 2A). 

#### 3.1.3. Larval Length

Growth was in part assessed by measuring the larval length and eye volume at 72 and 120 hpf. Larval length was measured from the tip of the head to the tip of the tail. Lengths of control (untreated) zebrafish were 3.35 ± 0.02 mm at 72 hpf and 3.79 ± 0.02 mm at 120 hpf; a growth rate of 0.44 mm over 48 h (Figure 2B). Exposure to sulfolane significantly reduced the larval length at 72 and 120 hpf (72 hpf: *p* < 0.0001; 120 hpf: *p* < 0.0001; Kruskal−Wallis test) and at concentrations greater than 1000 µg/mL, a significant difference from control was observed with a Conover–Iman all pairs comparison test (72 hpf: *p* < 0.00042; 120 hpf: *p* < 0.0001). The lowest larval length was observed at the maximum sulfolane exposure concentration tested (5000 µg/mL) for both 72 hpf (2.60 ± 0.05 mm) and 120 hpf (2.74 ± 0.05 mm).

#### 3.1.4. Larval Eye Volume

Eye volume was calculated by measuring the diameter under the assumption that eye is a sphere [35]. Control zebrafish larvae had an eye volume of 0.010 ± 0.0003 mm^3^ at 72 hpf and 0.014 ± 0.0004 mm^3^ at 120 hpf; an increase of 0.004 mm^3^ within 48 h (Figure 2C). Sulfolane exposure significantly reduced the eye volume compared to control (72 hpf: *p* < 0.0001; 120 hpf: *p* < 0.0001; Kruskal−Wallis test) at concentrations above 1000 µg/mL as determined by Conover–Iman all pairs comparison test (72 hpf: *p* < 0.00104; 120 hpf: *p* < 0.00336). The lowest eye volume was observed at the highest sulfolane concentration tested (5000 µg/mL) for both 72 hpf (0.006 ± 0.0002 mm^3^) and 120 hpf (0.006 ± 0.0004 mm^3^); indicated no growth.

#### 3.1.5. Yolk Sac and Pericardial Oedemas

**Figure 3:**
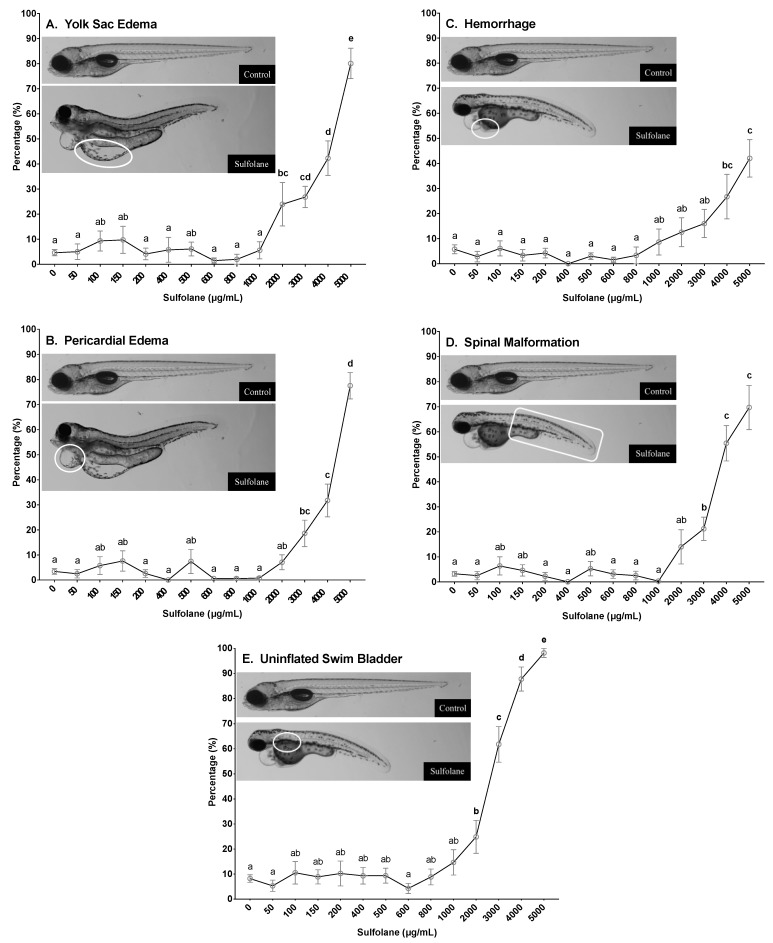
Dose-related effect of sulfolane exposure in µg/mL on the presence of yolk sac (**A**), pericardial oedemas (**B**), haemorrhage (**C**), spinal malformation (**D**), and uninflated swim bladder (**E**) in zebrafish (*Danio rerio*) larvae at 120 hpf. The points represent mean and SEM. Unmatched letters represent statistical significance (*p* < 0.05) based on tests with Conover–Iman all pairs comparison test. *n* = 8–15 wells, each containing 10 embryos.

Frequency of yolk sac and pericardial oedemas in surviving zebrafish larvae was quantified at 72 and 120 hpf by the presence of fluid in the yolk and pericardial cavity, respectively resulting in the distention of the cavities. Incidence of yolk sac and pericardial oedema at 120 hpf in control zebrafish larvae were 4.7 ± 1.2% and 3.4 ± 1.1% respectively. Exposure to sulfolane, significantly increased yolk sac oedemas (*p* < 0.0001; Kruskal−Wallis test) and at concentrations greater than 2000 µg/mL (24.0 ± 8.7%; Figure 3A), significant difference from control was observed (*p* < 0.00237; Conover–Iman all pairs comparison test). Exposure to sulfolane at 120 hpf also significantly increased pericardial oedemas (*p* < 0.0001; Kruskal−Wallis test) at concentrations greater than 3000 µg/mL (18.6 ± 5.3%; *p* < 0.0001, Conover–Iman all pairs comparison test; Figure 3B). The highest frequency was observed at the maximum exposure concentration of 5000 µg/mL for both yolk sac and pericardial oedemas at 120 hpf (yolk sac oedemas: 80.1 ± 6.1%; pericardial oedemas −77.6 ± 5.2%). Furthermore, using a nonlinear regression, the EC_50_ for yolk sac and pericardial oedemas were estimated to be 3883 µg/mL (CI: 3570–4249) and 4275 µg/mL (CI: 4104–4454), respectively at 120 hpf. Similar trends were also observed in yolk sac (*p* < 0.0001; Kruskal−Wallis test) and pericardial oedemas (*p* < 0.0001; Kruskal−Wallis test) at 72 hpf where significant differences were observed following sulfolane exposure at concentrations greater than 2000 µg/mL (*p* < 0.00336, Conover–Iman all pairs comparison test) and 3000 µg/mL respectively (*p* < 0.03885, Conover–Iman all pairs comparison test; supplementary data Figure S1A,B).

#### 3.1.6. Haemorrhaging

Frequency of haemorrhaging in surviving zebrafish larvae was characterised at 72 and 120 hpf by the pooling of blood in a tissue. Incidence of haemorrhaging at 120 hpf in control zebrafish larvae was 5.8 ± 1.8%. Exposure to sulfolane for 120 hpf, significantly increased the incidence of haemorrhaging (*p* < 0.0001; Kruskal−Wallis test) at concentrations higher than 4000 µg/mL (26.7 ± 8.9%; *p* < 0.02793, Conover–Iman all pairs comparison test; Figure 3C). The highest frequency of haemorrhaging was observed at the maximum concentration of 5000 µg/mL (42.0 ± 7.5%). No estimation of the EC_50_ for haemorrhaging was carried out due to the frequency at the highest concentration tested was less than 50%. Similar trends were also observed in haemorrhaging at 72 hpf (*p* < 0.0001; Kruskal−Wallis test). Exposure to sulfolane exposure, increased haemorrhaging and the differences became significant at the maximum sulfolane exposure concentration tested (5000 µg/mL; *p* < 0.00130, Conover–Iman all pairs comparison test; supplementary data Figure S1C).

#### 3.1.7. Spinal Malformations

Spinal malformation was characterised by the presence of lordosis, kyphosis or scoliosis. However, frequency of the different types of spinal malformations were not recorded and collectively referred to as spinal malformations. Incidence of spinal malformations at 120 hpf in control zebrafish larvae was 3.2 ± 1.0%. Exposure to sulfolane for 120 hpf, significantly increased spinal malformations (*p* < 0.0001; Kruskal−Wallis test) at concentrations higher than 3000 µg/mL (21.2 ± 4.6%; *p* < 0.0001, Conover–Iman all pairs comparison test; Figure 3D). The highest frequency of spinal malformations was observed at the highest concentration of sulfolane tested (5000 µg/mL; 69.7 ± 8.9%). Furthermore, using a nonlinear regression, the EC_50_ for the presence of spinal malformations following sulfolane exposure of 120 hpf was estimated to be 3936 µg/mL (CI: 3689–4223). A similar trend was also observed for spinal malformations at 72 hpf (*p* < 0.0001; Kruskal−Wallis test). However, significant difference from control was observed at sulfolane exposure concentrations greater than 4000 µg/mL (*p* < 0.0001, Conover–Iman all pairs comparison test; supplementary data Figure S1D).

#### 3.1.8. Swim Bladder Malformations

A swim bladder is a gas filled organ that aids in the neutral buoyancy of the fish, and is inflated by 120 hpf [46,47]. Zebrafish larvae that did not exhibit an inflated swim bladder at 120 hpf were recorded as having a swim bladder malformation. Incidence of swim bladder malformations at 120 hpf in control zebrafish larvae was 8.2 ± 1.5%. Exposure to sulfolane significantly increased swim bladder malformations (*p* < 0.0001; Kruskal−Wallis test) at concentrations greater than 2000 µg/mL (24.9 ± 6.6%; *p* < 0.0442, Conover–Iman all pairs comparison test; Figure 3E). The highest frequency of swim bladder malformations was observed at the highest concentration of sulfolane tested (5000 µg/mL; 98.2 ± 1.8%). Furthermore, using a nonlinear regression, the EC_50_ for the presence of swim bladder malformations following sulfolane exposure of 120 hpf was estimated to be 2581 µg/mL (CI: 2372–2776). 

### 3.2. Behavioural Assessment

#### 3.2.1. Response to Touch Stimuli

**Figure 4:**
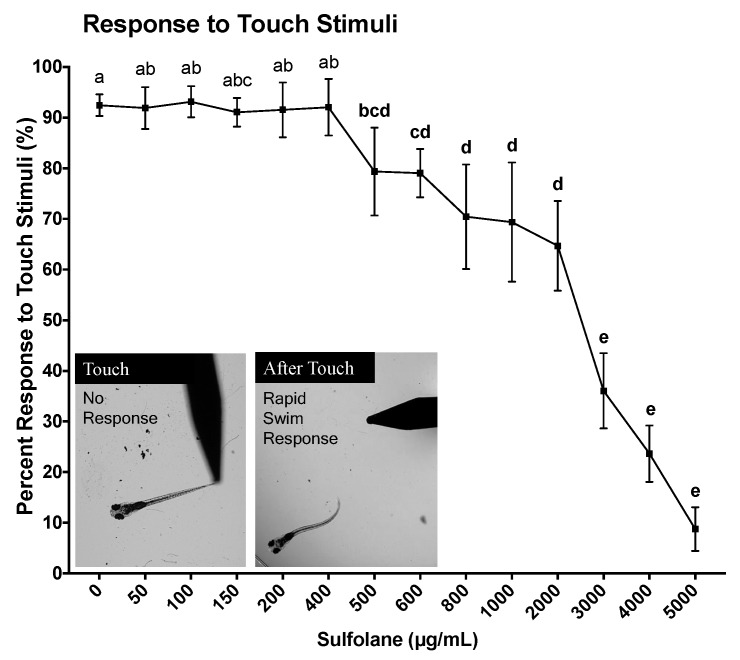
Dose-related effect of sulfolane exposure in µg/mL on the response to touch stimuli in zebrafish (*Danio rerio*) larvae at 120 hpf. The points represent mean and SEM. Unmatched letters represent statistical significance (*p* < 0.05) based on tests with Conover–Iman all pairs comparison test. *n* = 8–15 wells, each containing 10 embryos.

Response to touch stimuli was assessed at 120 hpf. Unanaesthetised larvae were touched at the tail to illicit a fast escape response following no sulfolane exposure (control untreated) or sulfolane exposure of varying concentrations (0–5000 µg/mL) for the duration of 120 hpf. No movement following touch was considered as an absence of touch response to stimuli. Untreated zebrafish larvae responded immediately to touch (92.5 ± 2.1%) by swimming away (Figure 4). Sulfolane exposure significantly reduced the response to touch stimulus (*p* < 0.0001; Kruskal−Wallis test) at concentrations higher than 500 µg/mL (*p* < 0.04556, Conover–Iman all pairs comparison test). At the highest sulfolane exposure concentration (5000 µg/mL), only 8.8 ± 4.3% zebrafish larvae were able respond to touch. Using a nonlinear regression, the EC_50_ for the lack of response to touch stimuli following sulfolane exposure of 120 hpf was estimated to be 1951 µg/mL (CI: 1598–2339).

#### 3.2.2. Locomotor Activity

**Figure 5:**
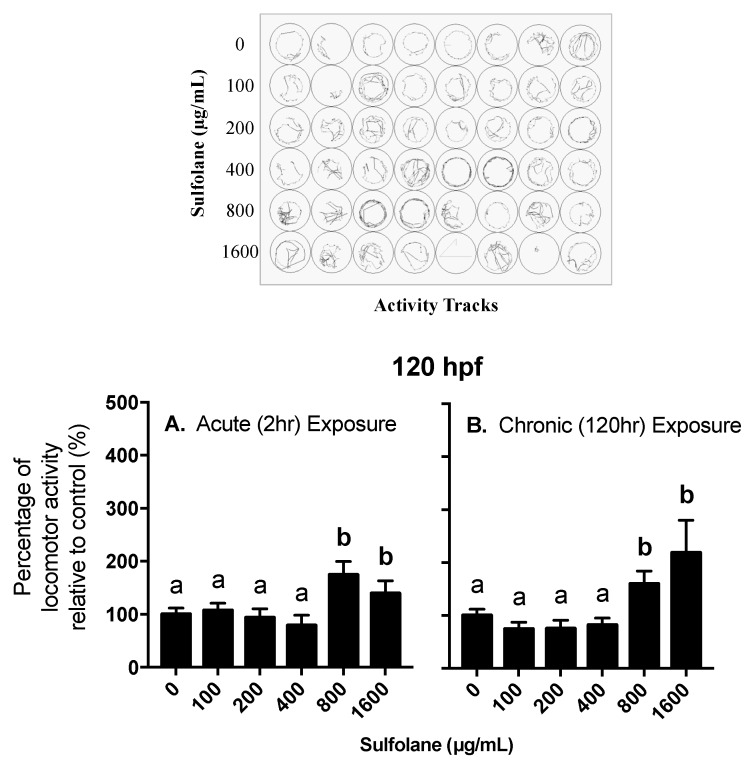
Dose-related effect of sulfolane exposure in µg/mL on the locomotor activity at dark / light stage of zebrafish (*Danio rerio*) larvae at 120 hpf. Results represent mean and SEM. Unmatched letters represent statistical significance (*p* < 0.05) based on tests with Conover–Iman all pairs comparison test. *n* = 30–40 individual larvae (**A**) Acute exposure of 2 h at 120 hpf timepoint (**B**) Chronic exposure of 120 h.

Locomotor behavioural response was investigated in 120 hpf control (no sulfolane exposure) zebrafish larvae following acute sulfolane exposure for 2 h as well as in 120 hpf zebrafish larvae that had a chronic sulfolane exposure for the duration of 120 h. Changes in locomotor activity (distance moved in a well of a 48-well plate) were measured in all groups during light/dark stimuli. A ratio of the total distance travelled while in the dark over the total distance travelled while in the light was calculated and normalised to 100% for control zebrafish larvae (0 µg/mL sulfolane exposure). Hyperactivity in the dark was observed following sulfolane exposure (acute: *p* = 0.0008309; chronic: *p* < 0.0001, Kruskal−Wallis test) at concentrations higher than 800 µg/mL for both acute (2 h; *p* < 0.00027, Conover–Iman all pairs comparison test) and chronic (120 hr; *p* < 0.0001, Conover–Iman all pairs comparison test) sulfolane exposures (Figure 5A,B). In all cases, larvae moved more in the dark than in light.

### 3.3. Transcript Abundance

In an attempt to gain an insight into the mechanisms of the adverse impacts of sulfolane, transcript abundance of various genes was quantified following exposure to varying concentrations of sulfolane for 120 hpf. Two sets of dose response experiments were carried out based on initial studies testing sulfolane concentrations between 125 and 4000 µg/mL. For transcripts displaying a significant difference from control at the lowest dose tested (125 µg/mL), we performed additional experiments expanding sulfolane concentrations ranging from 1 to 4000 µg/mL. 

#### 3.3.1. Aryl hydrocarbon receptor (*ahr1a*), Cytochrome p450 (*cyp1a*) and Heat shock protein (*hsp70*)

**Figure 6:**
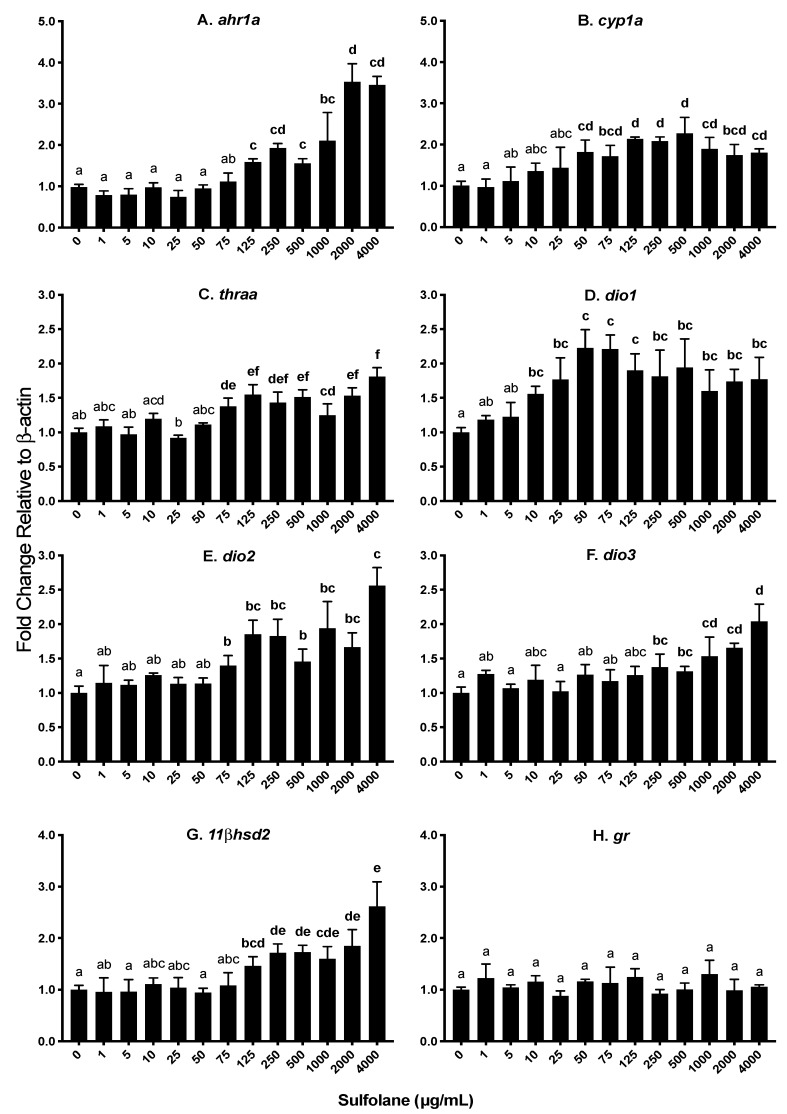
Transcript abundance expressed as a fold change relative to the house keeping gene *β-actin*. Results represent mean and SEM. Unmatched letters represent statistical significance (*p* < 0.05) based on tests with Conover–Iman all pairs comparison test; *n* = 4. (**A**) Aryl hydrocarbon receptor 1a (*ahr1a*); (**B**) cytochrome p450 1a (*cyp1a*); (**C**) thyroid receptor alpha (*thraa*); (**D–F**) deiodinase 1, 2 and 3 (*dio1*, *dio2* and *dio3*); (**G**) 11 beta – dehydrogenase type 2 (*11βhsd2*); and (**H**) glucocorticoid receptor (*gr*).

**Figure 7:**
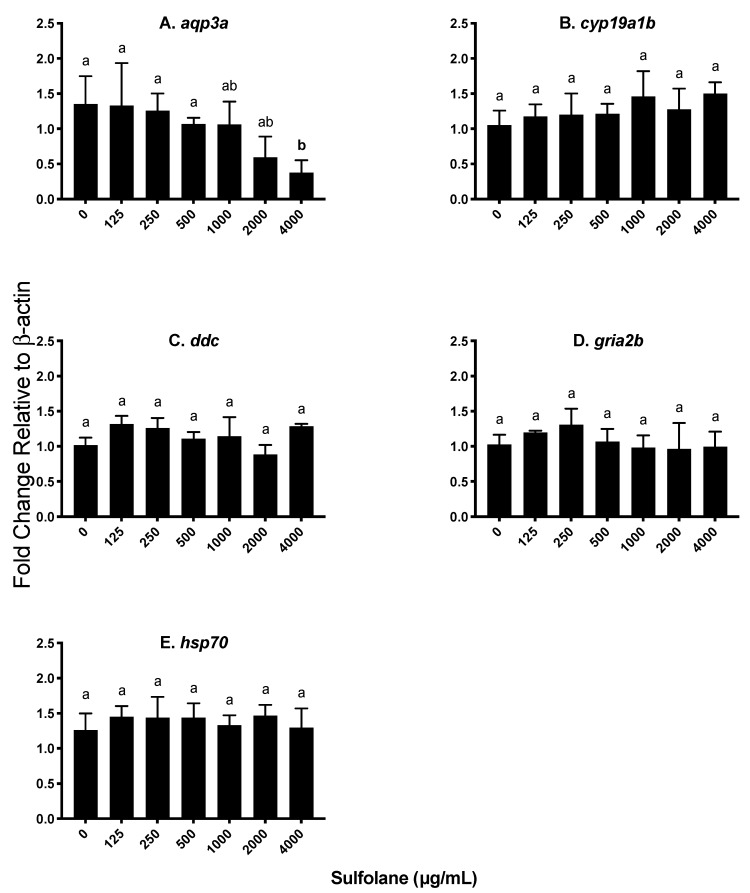
Transcript abundance expressed as a fold change relative to the house keeping gene *β-actin.* Results represent mean and SEM. Unmatched letters represent statistical significance (*p* < 0.05) based on tests with Conover–Iman all pairs comparison test; *n* = 4. (**A**) Aquaporin 3a (*aqp3a*), (**B**) brain aromatase (*cyp19a1b*), (**C**) dopa decarboxylase (*ddc*), (**D**) AMPA-type glutamate receptor (*gria2b*) and (**E**) heat shock protein 70 (*hsp70*).

Aryl hydrocarbon receptor type 1a (*ahr1a*) and cytochrome p450 1a (*cyp1a*) are involved in oxidative stress. Heat shock proteins are a protective mechanism to prevent denaturation of essential proteins from stressors such as oxidative stress. Exposure to sulfolane resulted in a significant increase in *ahr1a* levels (*p* = 0.0002373; Kruskal−Wallis test) at concentrations greater than 125 µg/mL (1.59 ± 0.01; *p* < 0.01213, Conover–Iman all pairs comparison test; Figure 6A). The highest expression of *ahr1a* was observed at the maximum sulfolane exposure concentration (4000 µg/mL). Aryl hydrocarbon receptor activation initiates *cyp1a* expression and as a result, expression of *cyp1a* was also quantified. Expression of *cyp1a* was found to increase following sulfolane exposure (*p* = 0.007216; Kruskal−Wallis test) at concentration higher than 50 µg/mL (1.82 ± 0.29; *p* < 0.00854, Conover–Iman all pairs comparison test; Figure 6B). Exposure to sulfolane was without effect (*p* = 0.4266; Kruskal−Wallis test) on heat shock protein 70 (*hsp70*) as represented in Figure 7E. 

#### 3.3.2. Thyroid hormone receptor (*thraa*) and Deiodinases 1, 2, and 3 (*dio1*, *dio2*, *dio3*)

Thyroid hormones are important in early embryonic development, and in this study transcript levels for thyroid hormone receptor (*thraa*), and deiodinases 1, 2 and 3 (*dio1*, *dio2* and *dio3*) were investigated. Deiodinases are enzymes involved in the regulation of activity of thyroid hormone. Exposure to sulfolane, significantly increased *thraa* (*p* = 0.0001994; Kruskal−Wallis test), *dio1* (*p* = 0.02751; Kruskal−Wallis test), *dio2* (*p* = 0.001712; Kruskal−Wallis test) and *dio3* (*p* = 0.02175; Kruskal−Wallis test). Increases in *thraa* were observed at concentrations higher than 75 µg/mL (1.38 ± 0.12; *p* < 0.00101, Conover–Iman all pairs comparison test; Figure 6C) while *dio1* and *dio2* transcript levels were increased at concentrations greater than 10 µg/mL (1.56 ± 0.11; *p* < 0.04016, Conover–Iman all pairs comparison test) and 75 µg/mL (1.40 ± 0.15; *p* < 0.02538, Conover–Iman all pairs comparison test) respectively (Figure 6D,E). Transcript levels of *dio3* increased at sulfolane concentrations greater than 250 µg/mL (1.38 ± 0.19; *p* < 0.04904, Conover–Iman all pairs comparison test). The increase in transcript levels of *dio1* plateaued at 50 µg/mL (Figure 6D), while the highest transcript abundance for *dio2* and *dio3* were observed at the highest sulfolane exposure concentration (4000 µg/mL; Figure 6E,F).

#### 3.3.3. 11 β Dehydrogenase (*11βhsd2*) and Glucocorticoid receptor (*gr*)

Stress plays an important role in embryonic development. 11 beta-dehydrogenase type 2 (*11βhsd2*) and glucocorticoid receptor (*gr*) are involved in the stress axis. Exposure to sulfolane significantly increased *11βhsd2* transcript levels (*p* = 0.002299; Kruskal−Wallis test) at concentrations higher than 125 µg/mL (1.46 ± 0.18; *p* < 0.03629, Conover–Iman all pairs comparison test; Figure 6G). The highest transcript abundance was observed at the highest sulfolane exposure concentration (4000 µg/mL). However, sulfolane exposure was without effect on *gr* transcript level (*p* = 0.6235; Kruskal−Wallis test; Figure 6H).

#### 3.3.4. Aquaporin (*aqp3a*)

Aquaporin 3a (*aqp3a*) is involved in the control of water permeability which may play a role in the presence of yolk sac and pericardial oedemas. Exposure to sulfolane significantly reduced *aqp3a* transcript levels (*p* = 0.0383; Kruskal−Wallis test) at the highest sulfolane exposure concentration tested (4000 µg/mL; *p* < 0.0469, Conover–Iman all pairs comparison test; Figure 7A). 

#### 3.3.5. Other Endocrine Parameters

The effect of sulfolane exposure was also investigated on genes encoding brain aromatase (*cyp19a1b*), dopa decarboxylase (*ddc*) and AMPA-type glutamate receptor (*gria2b*) as they are genes that may affect behaviour and other endocrine parameters. However, exposure to sulfolane was without effect on *cyp19a1b* (*p* = 0.1504; Kruskal−Wallis test), *ddc* (*p* = 0.3836; Kruskal−Wallis test) and *gria2b* (*p* = 0.2928; Kruskal−Wallis test) as represented in Figure 7B–D.

## 4. Discussion

Sulfolane is an emerging contaminant of concern due to its widespread presence in the environment in terrestrial and aquatic ecosystems. However, little is known about the impact of sulfolane exposure to organisms, especially to aquatic organisms. This study is the first to report the impact of sulfolane, apart from survival, on the embryonic development of an aquatic vertebrate model organism, zebrafish (*Danio rerio*). We used morphometric, behavioural and transcript abundance approaches to assess adverse health impact of sulfolane across a wide range of doses ranging from 1 to 5000 µg/mL. Lethal action of sulfolane was investigated and the results demonstrate 40% mortality at the highest concentration tested (5000 µg/mL). Statistically significant mortality was detected at concentrations above 800 µg/mL, which is a level found in the environment. However, the LC_50_ value was not calculated from the mortality curve since the highest concentration used had less than 50% mortality. 

Important morphological characteristics that affect survival and ability to swim in the wild include abnormally formed spine and improper swim bladder inflation. A normally formed spine allows for efficient swimming and escape from predators using the tail-flip response [48]. A tail-flip escape response is heavily reliant on the undulation of the tail to allow zebrafish to swim through the water [35,49]. Following sulfolane exposure, an increase in spinal malformations was observed making swimming difficult. This was exacerbated with a lack of normal swim bladder inflation following sulfolane exposure. Having a normal swim bladder helps with neutral buoyancy of the fish, minimising the energy cost of maintaining a constant depth in the water column [33]. This allows for a greater allocation of energy for growth, reproduction [50] and ability to escape from predators. Normal zebrafish larvae have an inflated swim bladder by 120 hpf [46,47], and lack of inflated swim bladder at 120 hpf would be a significant sign of larval malformation. A lack of swim bladder inflation is speculated to correlate with decreased survival in the wild.

Yolk sac and pericardial oedemas may also contribute to decreased survival by reducing swimming capability. Furthermore, previous studies have shown that fluid enter the yolk sac following contaminant exposure (10 ng/mL TCDD) may also disrupt osmoregulation [51,52,53,54]. Similarly, fluid can enter the pericardial cavity resulting in pericardial oedema. Thus, the observed increase in yolk sac and pericardial oedemas following sulfolane exposure may, in part, resulted from disruption in osmoregulation. This postulate is consistent with a previous observation where disruption of osmoregulation was linked to increase oedemas [51]. Additionally, exposure to contaminants such as sulfolane can result in the activation of detoxification mechanisms which can contribute to damaged endothelial cells, when coupled with the disruption of osmoregulation [55,56]. This is consistent with the observed increase in haemorrhaging following sulfolane exposure.

These pronounced malformations (spinal malformations, lack of swim bladder inflation, yolk sac oedema, pericardial oedema and haemorrhaging) pose significant challenges to growth due to the reallocation of energy resources to cope with the additional stress of a toxicant [57,58,59,60,61] and as such, growth was an important physiological and toxicology parameter measured. Larval length and eye volume were determined as direct assessment of growth, as well as yolk sac utilisation by measuring the yolk sac width as an indirect estimation of growth. In this context, increased growth requires the use of nutrients stored in the yolk sac and lower than normal yolk sac utilisation is related to impaired growth. In the present study yolk sac utilisation, larval length and eye volume have decreased by 120 hpf, indicating possibility of delayed development following sulfolane exposure. Previous studies have also demonstrated that contaminants may alter the rate of yolk sac utilisation and as such makes it a good indicator of delayed development [62,63,64].

Change in behaviour was used previously as a sublethal marker to assess neurotoxicity of chemicals [38,65,66]. Abnormal development of neural cells (through spinal malformations) can influence normal touch reflex and swim behaviour. Thus, a behavioural assessment was undertaken to determine if sulfolane affects response to touch stimuli and locomotion. Exposure to sulfolane significantly diminished response to touch stimuli in zebrafish larvae which can impair the ability of the larvae to escape predators and potentially reduce the fitness. Response to touch stimuli is in part dependent on the development of a small number of mechanoreceptors (neuromasts) within the mechanosensory lateral line developed by 72 to 96 hpf. These cells aid larvae in determining the orientation in water and avoidance of predators [67,68]. The results suggest that sulfolane may affect development of mechanoreceptors since at high concentrations fish totally stopped responding to touch stimuli. However, further study of mechanoreceptor development following sulfolane exposure will be needed to identify the exact developmental defects leading to impaired touch response.

Locomotor activity is also controlled by central nervous system and tracking of zebrafish larvae movement was studied following both chronic (120 h) and acute (2 h at 120 hpf) exposure to sulfolane. In the present study both chronic and acute exposures resulted in significant changes in locomotor activity in zebrafish larvae. This suggests that adverse effect can be observed after 2 h of exposure with sulfolane with similar sensitivity, although the magnitude of response was greater following chronic exposure. In this context, previous studies have shown changes in locomotor activity may be the result of developmental neurotoxicity [36,37,38], although there may be other contributing factors [69,70,71]. The present results are consistent with the previous studies, demonstrating increased locomotor activity (hyperactivity) following exposure to sulfolane in mouse and rat [6,17,18]. Furthermore, the results suggest a need to characterise the underlying reason for hyperactivity despite spinal malformations, and lack of response to touch stimuli.

To gain an insight into the mechanisms underlying the adverse effects of sulfolane, transcript abundance was quantified for a number of genes related to various endocrine and physiological functions in zebrafish embryos exposed to wide range of the contaminant concentrations. Previous studies demonstrated that hyperactivity may be related to hyperthyroidism [72] and as such transcript levels for genes related to thyroid hormone production and activity were measured. In the present study, transcript abundances of the thyroid receptor (*thraa*) and the deiodinases (*dio1*, *dio2* and *dio3*) were investigated. The enzymes, DIO1 and DIO2 are important for production of thyroid hormones (T_3_) from thyroxine (T_4_). Observed increase in *dio1* and *dio2* suggests sulfolane may lead to increased production of thyroid hormones. Stimulatory effects of sulfolane on *dio1* and *dio2* were observed at significantly lower concentration than that required to stimulate *dio3* which deactivates T_3_ by converting it to 3,5-diiodo-L-thyronine (T_2_) or T_4_ into reverse T_3_ [73]. Potentiation of T_3_ production by DIO1 and DIO2 further enhance thyroid activity since sulfolane also increased abundance of thyroid hormone receptor *thraa*. Thus, sulfolane appears to act as an endocrine-disrupting chemical by disrupting thyroid hormone axis in zebrafish larvae. Disruption of deiodinase production is considered to be a marker for thyroid hormone disruption [74,75]. Potential increase in thyroid axis could be a contributing factor in the observed hyperactivity. In this context, other studies demonstrated a link between hyperactivity and hyperthyroidism [72] as well as sulfolane and metabolic disruption in terrestrial organisms [76]. 

The impact of sulfolane exposure as a stressor was also investigated by measuring the transcript abundance of cortisol-responsive genes. Stress is mediated, in part, through the action of cortisol and an increase in cortisol levels has been attributed to an increase in locomotor activity in zebrafish [77]. Though the whole-body cortisol level was not quantified, transcript abundance of the 11 beta–hydroxysteroid dehydrogenase 2 (*11βhsd2*) gene was used as a predictor of cortisol level. Previous studies have shown correlation between increased cortisol levels and higher transcript abundance of *11βhsd2* [77,78]. In the present study, sulfolane exposure significantly increased transcript abundance of *11βhsd2* without affecting glucocorticoid receptor (*gr*). 

A previous study demonstrated that contaminants such as bisphenol-A can cause precocious neurogenesis leading to behavioural changes such as hyperactivity through an androgen and estrogen-dependent mechanism involving upregulation of aromatase [38]. In the present study, however, aromatase (*cyp19a1b*) transcript level did not change following exposure with sulfolane in zebrafish embryos. Dopa decarboxylase (*ddc*) is involved in the synthesis of dopamine and serotonin which are related to movement disorder as well as a number of other functions [79]. However, sulfolane exposure did not affect *ddc* transcript abundance in zebrafish larvae. Similarly, exposure to sulfolane did not influence AMPA-type glutamatergic receptor (*gria2b*) abundance, which is known to exist in zebrafish early in development and mediate response to touch stimuli (24 hpf) [80]. Behaviour is under multifactorial control [36,37,38,70,71,81], and changes in thyroid hormone activity and stress response may be contributing factors mediating hyperactivity as suggested previously [72,76,77].

Oedemas were also observed following exposure to sulfolane. Aryl hydrocarbon receptor (*ahr1a*) and cytochrome p450 (*cyp1a*) are involved in the response to contaminants causing oxidative stress. Aryl hydrocarbon receptor is a cytosolic, ligand-activated transcription factor that activates cytochrome p450 monooxygenase which is a detoxification enzyme, catalysing oxidative reactions [82,83]. Previous studies with contaminants, 2,3,7,8-tetrachlorodibenzo-p-dioxin (TCDD) and 3,3’,4,4’,5-pentachlorobiphenyl (PCB126) provided evidence for a link between the induction of *cyp1a* through *ahr1a* and yolk sac oedema, pericardial oedemas and spinal malformations [51,84,85]. This is consistent with the observed increase in transcript abundance of *ahr1a* and *cyp1a* following exposure to sulfolane. 

Additionally, when organisms are exposed to stressors, protective mechanisms such as heat shock proteins are activated which ensure maintenance of the structure of proteins and reduce apoptosis. Lack of an increase in protective mechanism of heat shock proteins results in an increase in protein denaturation leading to proteotoxicity [86] and eventual mortality. Heat shock protein 70 (*hsp70*) is highly conserved within vertebrates and is expressed early in zebrafish development (48–72 hpf) [87,88,89]. Heat shock protein 70 was shown to exert protective action against chemical stressors [86,89,90]. In the present study, exposure to sulfolane did not alter *hsp70* transcript abundance, suggesting that chemical stress response may have contributed to adverse health effects and mortality in zebrafish.

Osmoregulation is controlled by the osmotic gradient established by the gills and digestive system until the kidneys are functional [51,91,92]. These organs contain aquaporin channels which play an important role in regulating water and ion balance across cell membranes. Previous research have shown *aqp3a* expression decreasing following movement of fish from freshwater (hypotonic) to seawater (hypertonic) [93,94] to maintain osmotic balance. Exposure to sulfolane reduced, *aqp3a* transcript abundance at concentrations that caused pericardial and yolk sac oedema. The results suggest that reduction in *aqp3a* expression may be a contributing factor leading to oedema in developing zebrafish.

Overall, with three approaches in this study (morphometric, behavioural and transcript abundance), the results provide a spectrum of dose-related effects for the parameters measured. Increased mortality was observed above 800 µg/mL but deformities in growth were only observed at concentrations above 1000 µg/mL, and morphometric malformations at 3000 µg/mL. Furthermore, differences in behaviour and gene expression were observed at concentrations above 500 µg/mL and 10 µg/mL, respectively. The observed differences in the sensitivities to the sublethal effects of sulfolane facilitate assessment of the health impact of sulfolane in the model organism used. In this context, the present study revealed that changes in transcript abundance and behaviour can be seen, at doses much smaller than those causing changes in growth and mortality [95].

## 5. Conclusions

This study is the first to report the impact of sulfolane exposure on the development of zebrafish embryos utilising three different experimental approaches, including morphometrics, behavioural and transcript abundance. The results suggest that zebrafish embryo may be used as a suitable model organism to assess toxicity of sulfolane which is used extensively around the world. The findings provide evidence that sulfolane exert adverse effects with different sensitivity depending on the parameters measured in the developing zebrafish embryos, and a framework for better understanding of sulfolane toxicity in vertebrates in general. 

## Figures and Tables

**Table 1:** Comparison between the nominal sulfolane concentration and the measured sulfolane concentration in the 12 well plate following 24-h incubation of 96 hpf zebrafish (*Danio rerio*) larvae (96–120 hpf). The values are represented as mean ± SEM. *n* = 5 wells.

Nominal Concentration (µg/mL)	Measured Concentration at 120 hpf (µg/mL)
0	0 ± 0
50	49.90 ± 1.21
500	503.39 ± 4.13
5000	4950.36 ± 25.98

## Supplementary Materials

The following are available online at https://www.mdpi.com/2305-6304/7/3/42/s1, Figure S1: Dose-related effect of sulfolane exposure in µg/mL on the presence of various characterized morphometric endpoints in zebrafish (Danio rerio) larvae at 72 hpf; Table S1: List of genes used for exploratory transcript abundance assessment, their associated forward and reverse primers and their annealing temperature.
