# Supplementary material for: Health Impact Assessment of Sulfolane on Embryonic Development of Zebrafish (Danio rerio)
Source: Toxics. 2019 Aug 23;7(3):42. doi: 10.3390/toxics7030042 (PMC6789604; doi:10.3390/toxics7030042)
Supplement: Supplementary file 1 [file toxics-07-00042-s001.pdf]

Supplementary Materials: Health Impact Assessment of Sulfolane on Embryonic Development of Zebrafish *(Danio rerio)*

Soham M. Shah, Michael Wahba, Linlong Yu, Gopal Achari and Hamid R. Habibi

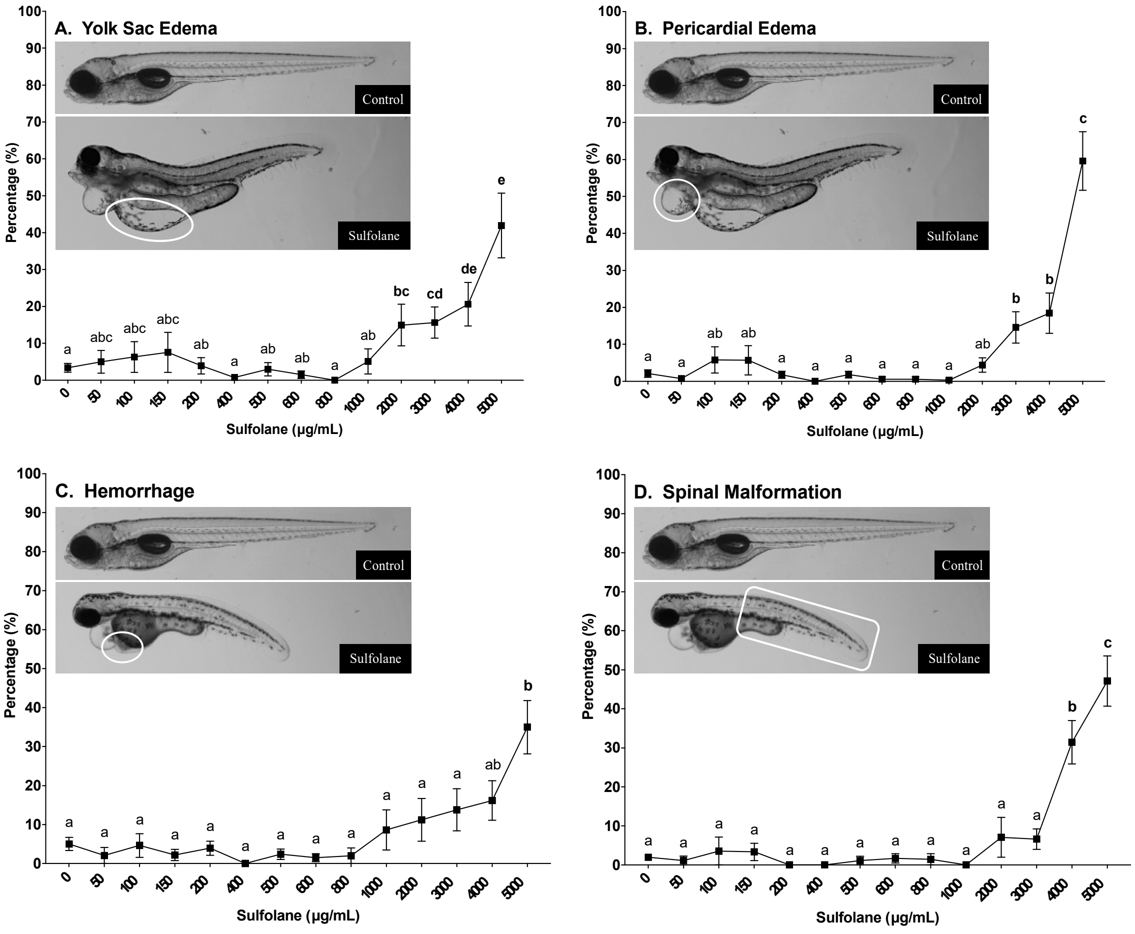

Figure S1. Dose-related effect of sulfolane exposure in µg/mL on the presence of various characterized morphometric endpoints in zebrafish *(Danio rerio)* larvae at 72hpf. The points represent mean and SEM. Unmatched letters represent statistical significance ($p < 0.05$) based on results of Conover-Iman all pairs comparison test. n = 6–10 wells, each containing 10 embryos. (**A**) Yolk Sac Oedema (**B**) Pericardial Oedema (**C**) Hemorrhaging (**D**) Spinal Malformations.

Table S1. List of genes used for exploratory transcript abundance assessment, their associated forward and reverse primers and their annealing temperature.

Gene	Type	Sequence	Tm (°C)	Reference
ß-actin	Forward	5′ – GCAGAAGGAGATCACATCCCTGGC – 3′	66.7	[1]
	Reverse	5′ – CATTGCCGTCACCTTCACCGTTC – 3′		
ahr1a	Forward	5′ – CGCAAAAGGAGGAAACCTGTC – 3′	52.8	[2]
	Reverse	5′ – CCTGTAGCAAAAATTCCCCCT – 3′		
cyp1a	Forward	5′ – ATTCATCCTTCCTTCCCTTCAC – 3′	56.0	[3]
	Reverse	5′ – ACCTTCTCGCCTTCCAACTTAT – 3′		
thraa	Forward	5′ – CTGCTGTGATTGGATGCTGGAT – 3′	51.0	[4]
	Reverse	5′ – GCTCTTGTCGGATGTGCTGTT – 3′		
dio1	Forward	5′– TGCTTTAATTACCCTGGACCG – 3′	55.0	Habibi Lab
	Reverse	5′ – TGCTGAAGTCCTTGACAAGC – 3′		
dio2	Forward	5′ – GCATAGGCAGTCGCTCATTT – 3′	54.0	[5]
	Reverse	5′ – TGTGGTCTCTCATCCAACCA – 3′		
dio3	Forward	5′ – TCCGACAGCAACAAGATGTTCACG – 3′	55.0	Habibi Lab
	Reverse	5′ – GCGCTCTTGAAGAAGTCCAGCTT – 3′		
11βhsd2	Forward	5′ – TGCTGCTGGCTGTACTTCAC – 3′	56.6	[6]
	Reverse	5′ – TGCATCCAACTTCTTTGCTG – 3′		
gr	Forward	5′ – ACAGCTTCTTCCAGCCTCAG – 3′	55.5	[6]
	Reverse	5′ – CCGGTGTTCTCCTGTTTGAT – 3′		
aqp3a	Forward	5′ – TGGACCCCTACAACAACCCG – 3′	59.9	[7]
	Reverse	5′ – TGCCATCCCACCATCAGC – 3′		
cyp19a1b	Forward	5′ – TCGGCACGGCGTGCAACTAC – 3′	59.0	[8]
	Reverse	5′ – CATACCTATGCATTGCAGACC – 3′		
ddc	Forward	5′ – CTGAGGAGGCCCCGGAGGAG – 3′	55.5	[9]
	Reverse	5′ – GGGCTGTGCCAGTGGGTGAC – 3′		
gria2b	Forward	5′ – CGCGCTCAGCCTGAGCAATGT – 3′	66.7	[10]
	Reverse	5′ – GGATATCAGCTCGGTAAGCGAGTGTC – 3′		
hsp70	Forward	5′ – ATCCTCATGGGCGACACCTCTG – 3′	58.0	Habibi Lab
	Reverse	5′ – TGTCGGCCTCTTGCACCATTCTC – 3′		

References

1. Casadei, R.; Pelleri, M.C.; Vitale, L.; Facchin, F.; Lenzi, L.; Canaider, S.; Strippoli, P.; Frabetti, F. Identification of housekeeping genes suitable for gene expression analysis in the zebrafish. *Gene Expr. Patterns* **2011**, *11*, 271–276.
2. Goodale, B.C.; la Du, J.K.; Bisson, W.H.; Janszen, D.B.; Waters, K.M.; Tanguay, R.L. AHR2 mutant reveals functional diversity of aryl hydrocarbon receptors in zebrafish. *PLoS ONE* **2012**, *7*, e29346.
3. Voelker, D.; Vess, C.; Tillmann, M.; Nagel, R.; Otto, G.W.; Geisler, R.; Schirmer, K.; Scholz, S. Differential gene expression as a toxicant-sensitive endpoint in zebrafish embryos and larvae. *Aquat. Toxicol.* **2007**, *81*, 355–364.
4. Huang, S.S.Y.; Benskin, J.P.; Veldhoen, N.; Chandramouli, B.; Butler, H.; Helbing, C.C.; Cosgrove, J.R. A multi-omic approach to elucidate low-dose effects of xenobiotics in zebrafish (*Danio rerio*) larvae. *Aquat. Toxicol.* **2017**, *182*, 102–112.
5. Zhai, W.; Huang, Z.; Chen, L.; Feng, C.; Li, B.; Li, T. Thyroid endocrine disruption in zebrafish larvae after exposure to mono-(2-ethylhexyl) phthalate (MEHP). *PLoS ONE* **2014**, *9*, e92465.
6. Alsop, D.; Vijayan, M.M. Development of the corticosteroid stress axis and receptor expression in zebrafish. *Am. J. Physiol. Regul. Integr. Comp. Physiol.* **2008**, *294*, R711–R719.

7. Horng, J.L.; Chao, P.L.; Chen, P.Y.; Shih, T.H.; Lin, L.Y. Aquaporin 1 is involved in acid secretion by ionocytes of zebrafish embryos through facilitating CO_2 transport. *PLoS ONE* **2015**, *10*, 0136440.
8. Brion, F.; Le Page, Y.; Piccini, B.; Cardoso, O.; Tong, S.-K.; Chung, B.; Kah, O. Screening estrogenic activities of chemicals or mixtures in vivo using transgenic (cyp19a1b-GFP) zebrafish embryos. *PLoS ONE* **2012**, *7*, e36069.
9. Pavlidis, M.; Sundvik, M.; Chen, Y.C.; Panula, P. Adaptive changes in zebrafish brain in dominant-subordinate behavioral context. *Behav. Brain Res.* **2011**, *225*, 529–537.
10. Lin, W.-H.; Wu, C.-H.; Chen, Y.-C.; Chow, W.-Y. Embryonic expression of zebrafish AMPA receptor genes: Zygotic gria2alpha expression initiates at the midblastula transition. *Brain Res.* **2006**, *1110*, 46–54.
